# Molecular Rapid Test for Identification of Tuna Species

**DOI:** 10.3390/bios14020082

**Published:** 2024-02-02

**Authors:** Isidora P. Gkini, Panagiotis Christopoulos, Alexis Conides, Despina P. Kalogianni, Theodore K. Christopoulos

**Affiliations:** 1 Analytical/Bioanalytical Chemistry & Nanotechnology Group, Department of Chemistry, University of Patras, 26504 Patras, Greece; isidgini@yahoo.gr (I.P.G.); pkchristop@gmail.com (P.C.); 2 Hellenic Centre for Marine Research, Institute for Marine Biological Resources, 46.7 km Athens-Sounion, Mavro Lithari, Anavyssos, 19013 Attika, Greece; conides@hcmr.gr; 3 Institute of Chemical Engineering Sciences/Foundation for Research and Technology Hellas (FORTH/ICE-HT), 26504 Patras, Greece

**Keywords:** bluefin tuna species, *Katsuwonus pelamis*, *Thunnus albacares*, *Thunnus thynnus*, fish adulteration, fish authentication, biosensor, gold nanoparticles, rapid test, DNA

## Abstract

Tuna is an excellent food product, relatively low in calories, that is recommended for a balanced diet. The continuously increasing demand, especially for bluefin-tuna-based food preparations, and its relatively high market price make adulteration by intentionally mixing with other lower-priced tunas more prospective. The development of rapid methods to detect tuna adulteration is a great challenge in food analytical science. We have thus developed a simple, fast, and low-cost molecular rapid test for the visual detection of tuna adulteration. It is the first sensor developed for tuna authenticity testing. The three species studied were *Thunnus thynnus* (BFT), *Thunnus albacares*, and *Katsuwonus pelamis*. DNA was isolated from fresh and heat-treated cooked fish samples followed by PCR. The PCR products were hybridized (10 min) to specific probes and applied to the rapid sensing device. The signal was observed visually in 10–15 min using gold nanoparticle reporters. The method was evaluated employing binary mixtures of PCR products from fresh tissues and mixtures of DNA isolates from heat-treated tissues (canned products) at adulteration percentages of 1–100%. The results showed that the method was reproducible and specific for each tuna species. As low as 1% of tuna adulteration was detected with the naked eye.

## 1. Introduction

Food authenticity is an important issue for consumers, food manufacturers, and regulatory bodies, as it directly impacts food safety, consumers’ trust, and fair-trade practices. Since there is no international regulation in place to control and detect adulteration, food fraud and adulteration comprise major challenges in the food sector [1]. Tuna is one of the most widely consumed fish species worldwide. It is a highly nutritious food that is rich in protein, omega-3 fatty acids, and other essential nutrients. Tuna is also a versatile food that can be prepared in a variety of ways, including canned, fresh, or frozen products, and is used in a wide range of dishes such as salads, sandwiches, sushi, and pasta. In addition to its nutritional value, tuna is also an important economic resource for many countries [1,2,3]. To commemorate the significance of tuna fish species and the difficulties facing tuna stocks worldwide, the United Nations declared May 2nd as ‘World Tuna Day’. The sustainability of tuna stocks is threatened by several factors, including overfishing, illegal fishing, and climate change. World Tuna Day provides a chance to draw a spotlight on the need for action to guarantee the long-term existence of these species [2]. The mislabeling of tuna species could hide market biodiversity and result in the unintentional food intake of adulterant species [3]. As a result, the development of fast and user-friendly methods to identify tuna adulteration remains challenging in the field of food analytical science.

The existing methods to determine food authenticity are morphology-based approaches, ingredient-targeted analyses, spectroscopic methods, chromatography, and protein-based methods [4,5,6]. Spectroscopic techniques coupled with chemometrics and molecular methods are the most widely used for fish authenticity testing. As for protein-based techniques, these include proteomics based on mass spectrometry [7,8,9,10,11,12,13], two-dimensional electrophoresis with subsequent analysis with mass spectrometry [14], isoelectric focusing [15,16], enzyme-linked immunosorbent assay (ELISA) [17,18,19,20], and capillary electrophoresis [21]. The methods developed for the identification of individual species are time-consuming and less successful in processed foods since the specific morphological traits are transformed throughout the processing. Also, they require sophisticated equipment and highly qualified personnel [22]. On the other hand, fish and seafood fraud has been effectively detected using DNA fingerprinting. Because of its higher stability in a variety of matrices and conditions, DNA, as a marker, has the advantage of enabling species identification in either fresh or processed samples of fish and seafood [1]. All molecular methods that have been developed to identify tuna species include an exponential amplification step of characteristic DNA sequences mainly with PCR [23,24,25,26,27,28,29,30,31,32,33] but also with LAMP [3,34]. The detection of the amplification products is commonly based on the principle of fluorescence resonance energy transfer (FRET), in which a specialized and costly oligonucleotide probe, double labeled with a fluorophore and a quencher, is hydrolyzed, during the extension step of each cycle, by a specialized DNA polymerase, which also possesses 5′ > 3′ exonuclease activity, resulting in an increase in the distance between the fluorophore and quencher with a concomitant increase in fluorescence intensity [23,25]. In another report, the double labeled probe has a stem-loop structure (molecular beacon) that opens upon hybridization with the amplified target sequences, and as a result, the distance between fluorophore and quencher increases [34]. A dsDNA-specific fluorescent dye has also been used [3], but it has the disadvantage that fluorescence is obtained from any non-specific dsDNA products, thus increasing the probability of false positive results. Alternatively, the target sequences were labeled with a hapten during amplification, then purified and detected by an enzyme-conjugated anti-hapten antibody in combination with a suitable substrate, a procedure that requires several incubation and washing steps [32]. Furthermore, electrophoresis has been used for size separation and detection of the amplification products from tuna species [24]. Other methods have been based on next-generation sequencing [33], and the performance of a primer extension reaction on the PCR product followed by sequencing [31].

Currently, there is a pressing challenge for the development of fish authenticity tests, which could potentially be applied on-site. Towards this goal, in the present work, we propose a novel method based on a simple and portable, strip-type user-friendly sensor for rapid, visual molecular testing of tuna species without the need for sophisticated instrumentation and fluorophore-labeled probes. A short hybridization step (10 min) of the amplified sequences with a specific unconjugated oligonucleotide probe, inside the thermal cycler, follows the PCR, and then the unpurified product is placed on the DNA-sensing device, which provides a visual signal within 10–15 min if the tuna species is present in the sample. The reagents required for detection are deposited, in dry form, or immobilized on the sensing device to minimize the need for transport and storage and to avoid multiple pipetting steps. The method was assessed with three species, that is, *Katsuwonus pelamis* (skipjack tuna), *Thunnus albacares* (yellowfin tuna), and *Thunnus thynnus* (bluefin tuna).

## 2. Experimental Section

### 2.1. Materials

DNA isolation was performed by using the NucleoSpin Tissue extraction DNA kit from Macherey-Nagel (Düren, Germany). The nitrocellulose membrane Immunopore FP, blotting papers, and glass fiber Standard 17 conjugation pad for the construction of the strips were from Cytiva (Marlborough, MA, USA). Sucrose was from AppliChem (Maryland Heights, MO, USA), while methanol, hydrochloric acid, sodium azide, EDTA, and sodium hydroxide were from Merck (Darmstadt, Germany). Borax, sodium dodecyl sulfate (SDS) and anti-biotin antibody produced in goat were purchased from Sigma-Aldrich (St. Louis, MO, USA). Albumin bovine fraction V, pH 7.0, standard grade, lyophilized, was from Serva Electrophoresis (Duisburg, Germany), Tris-base from Fischer Scientific (Waltham, MA, USA), glycerol from Lachner (Neratovice, Czech Republic), and Tween-20 from Fluka (Paris, France). Unconjugated gold colloid, 40 nm in diameter, was obtained from BBI Solutions (Crumlin, UK), the Kapa2G Fast PCR Kit was from Κapa Biosystems (Wilmington, MA, USA), dNTPs from Invitrogen (CarlsBad, CA, USA), and terminal transferase from New England Biolabs (Ipswich, MA, USA). The primers and probes were purchased by Eurofins Genomics (Ebersberg, Germany), and the sequences are presented in Table 1. The 1×Phosphate-buffered saline (PBS) solution consisted of 137 mM NaCl, 2.7 mM KCl, 8 mM Na_2_HPO_4_, and 2 mM KH_2_PO_4_, adjusted to pH 7.4. The 6×Saline-Sodium Citrate (SSC) buffer consisted of 900 mM sodium chloride and 90 mM sodium citrate and was adjusted to pH 7.0.

### 2.2. Apparatus

The spectrophotometer DS-11 from DeNovix (Wilmington, DE, USA) was used for the quantification of the isolated DNA, while PCR was performed in the MiniTurbo Portable Thermal Cycler from Blue-Ray Biotech (Taiwan). The automated dispenser Linomat 5 from Camag (Muttenz, Switzerland) and the UVP Crosslinker CL-3000 from Analytik Jena (Upland, CA, USA) were used for the construction of the sensing zones of the rapid tests, and an Epson Perfection V600 Photo Scanner (Seiko Epson Corporation, Suwa, Japan) was employed for image acquisition of the rapid tests.

### 2.3. Fish Samples

The fish samples used for this study were fresh fish tissues and heat-treated cooked fish samples simulating the canned conditions from different tuna species. Three tuna species were examined: *K. pelamis*, *T. albacares*, and *T. thynnus*. The *T. albacares* and *K. pelamis* samples were fished from the Pacific Ocean, while the *T. thynnus* was from the Ionian Sea.

### 2.4. Preparation of PolydA-Probe and PolydT-dT_30_ Probe: Tailing Reaction

Deoxynucleotides were added to the 3’ hydroxyl end of each specific probe (ALBP, PELP, THYP) for each tuna species and the dT_30_ probe via a terminal transferase reaction. A polydA and polydT tail was added to the specific probes and the dT_30_ probe, respectively. The reaction mixture contained 1×TdT Buffer, 0.25 mM CoCl_2_, 2 mM dATP for polyA tail or dTTP for the polyT tail, 0.02 mM of each probe, and 20 U Terminal Transferase. The mixture was diluted with deionized water to the final volume of 20 μL and incubated at 37 °C for 1 h. Finally, the reaction was terminated by adding 2 μL of 0.5 M EDTA, pH 8.0. The final concentration of the polydA-probe was 18.2 μmol/L, and for the polydT-dT_30_ probe, it was 45.5 μmol/L.

### 2.5. Statistical Methods

Means were examined for precision and repeatability using the coefficient of variation:

$$\%CV=\frac{standard\;deviation}{mean}\times 100.$$

Values of %CV below 5% indicate high precision and repeatability [19].

## 3. Results and Discussion

**Figure 1:**
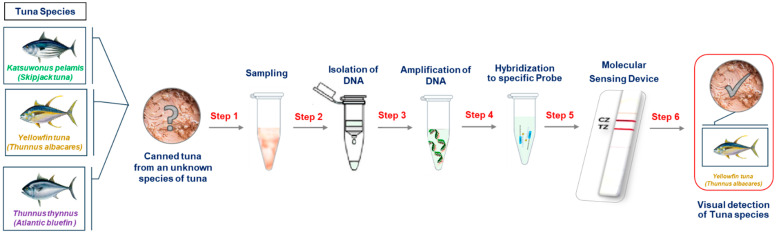
A step-by-step guide to the visual distinction of tuna species.

In this study, we have developed, for the first time, a new molecular rapid test for fish species authentication. As a model, three of the most common tuna fish species were detected and identified with the naked eye using the molecular rapid test. Yellowfin tuna (*T. albacares*), Atlantic Bluefin tuna (*T. thynnus*), and Skipjack tuna (*K. pelamis*) species were selected for this study. The method included the following steps: (i) DNA extraction from fish tissue (1.5–2.5 h), (ii) PCR in a mini portable thermocycler (70–80 min), (iii) hybridization of the amplification products to species-specific polydA-probes (15 min) and (iv) visual detection and simultaneous identification of the tuna species with the sensing device (10 min) (Figure 1).

**Figure 2:**
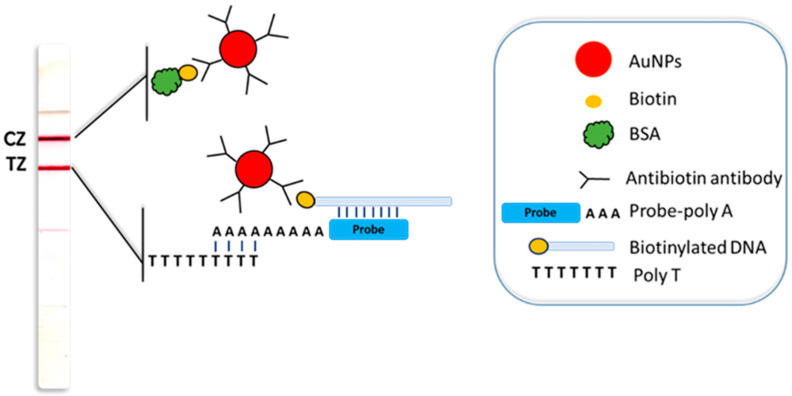
Schematic illustration of the molecular rapid test. AuNPs: gold nanoparticles; BSA: bovine serum albumin; TZ: test zone; CZ: control zone.

The developed molecular rapid test was applied to lateral muscle tissue samples from fresh fish and heat-treated cooked fish. Figure 2 illustrates the principle of the molecular rapid test. Briefly, DNA was extracted from fish tissue (fresh or cooked) and subjected to PCR using species-specific primers. The amplified sequences were labeled with biotin through a biotinylated primer. Τhe product sizes were: 85 bp for *T. albacares*, 200 bp for *T. thynnus*, and 238 bp for *K. pelamis*. A typical electropherogram of the three PCR products for the three tuna species in 2% agarose gel with ethidium bromide staining is presented in Figure S1 (Supporting Information).

The experimental biotinylated PCR products were hybridized to species-specific probes that carried a polydA tail. The hybrids were applied to the conjugate pad of the sensor, and the strip was dipped into a proper developing solution. As the solution moved upwards, the DNA sample was mixed with the nanoparticles. A first red line was formed as the hybrids were captured by the immobilized polydT probe at the test zone of the strip through hybridization with the polydA tail of the probe, and gold nanoparticles were accumulated through anti-biotin antibody interaction with the biotin moieties of the PCR product. A second red line was also formed, afterwards, as the excess of the nanoparticles were captured by immobilized biotinylated BSA at the control zone of the test, ensuring that the process of nanoparticle redispersion and flow was efficient.

Studies in this work included the optimization of the DNA isolation procedure, the PCR, the hybridization of the PCR products to the species-specific probes, the detectability of the method, that is, the lowest concentration for each tuna species that can be visualized by the device, the specificity and the precision of the method, as well as the performance of the method when applied to real samples for the detection of fish adulteration in heat-processed fish mixtures of different tuna species.

### 3.1. Optimization Studies

Initially, various DNA isolation kits were tested to select the optimum that gives the best yield. DNA extraction was performed from fresh samples using three commercial kits: NucleoSpin Tissue (Macherey-Nagel), NucleoSpin Plant II (Macherey-Nagel), and the Genomic DNA Isolation Kit from Norgen Biotek. The results from the absorbance measurements at 260 nm are presented in Table S1. The NucleoSpin Tissue kit provided the highest concentrations of isolated DNA for all three tuna species.

Then, the annealing temperature of the primers for PCR was optimized. Three different annealing temperatures (50, 55, and 62 °C) were assessed using DNA isolated from fresh *K. pelamis* tissue. The PCR products were then separated by electrophoresis in 2% agarose gel using ethidium bromide for staining. The electropherogram is presented in Figure S2 (Supporting Information). The results from the electrophoresis, after densitometric analysis of the zones using the Image J software 1.54g National Institutes of Health (NIH), Bethesda, MD, USA and Laboratory for Optical and Computational Instrumentation, University of Wisconsin, Madison, WI, USA), showed that the yield of PCR was higher when the annealing temperature was set at 62 °C. The PCR cycles were also optimized. A constant amount of DNA (100 ng) was subjected to 25, 30, and 35 cycles of PCR. As observed in Figure S3, no PCR product was produced at 25 cycles, while 30 and 35 cycles provided efficient PCR yield.

Afterwards, the hybridization reaction of the PCR products to the species-specific probe was also optimized. Two hybridization temperatures, 37 °C and 42 °C, were assessed for the hybridization of 100 fmol of PCR product for *K. pelamis* to its specific probe prior to the application to the sensing device. The results are shown in Figure S4. No significant difference in the signal between the two hybridization temperatures was observed. However, the temperature of 37 °C was chosen, as the signal at the test zone of the strip was slightly stronger.

The concentration of species-specific probes in the hybridization reaction was also optimized. For this purpose, an amount of 6.3 fmol of PCR product of *K. pelamis* was hybridized to its specific probe in three different concentrations: 0.5, 1, and 2 μM. As observed in Figure S5, the optimum concentration of the specific probe is 0.5 μM, because it provided the most intense signal at the test zone of the sensor.

### 3.2. Effect of the Amount of Amplification Product on the Visual Signal

**Figure 3:**
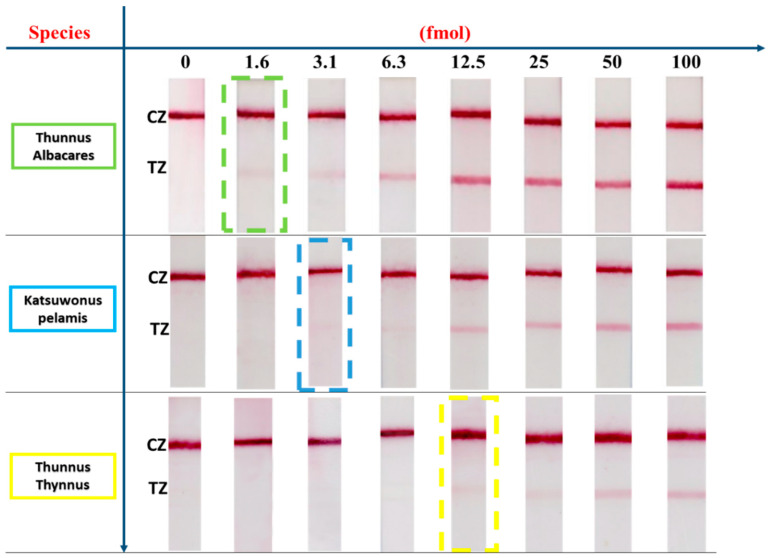
Effect of the amount of amplification product on the signal of the sensor for the three species. The amount of PCR product ranged from 1.6 to 100 fmol. TZ: test zone; CZ: control zone. The dashed boxes correspond to the detectability of the sensor for each fish species.

The effect of the amount of the amplification product on the signal of the sensor for each species was studied by preparing dilutions of each PCR product derived from fresh tissue and applying to the sensors various amounts of the PCR product ranging from 1.6 to 100 fmol. A sample that contained no DNA target was also included in the study as a negative control to confirm the absence of false positive signals. The results for all three tuna species are presented in Figure 3.

Based on Figure 3, the detectability of the method varied according to the tuna species. We were able to detect as low as 1.6, 3.1, and 12.5 fmol of PCR product for *T. albacares*, *K. pelamis*, and *T. thynnus*, respectively, with the naked eye using the proposed sensing device. No false positive signals were observed.

### 3.3. Specificity

The specificity of the method is based on the ability of the oligonucleotide probes to recognize only their cognate target sequences. Any cross-hybridization between the probes and targets would lead to false positive results.

**Figure 4:**
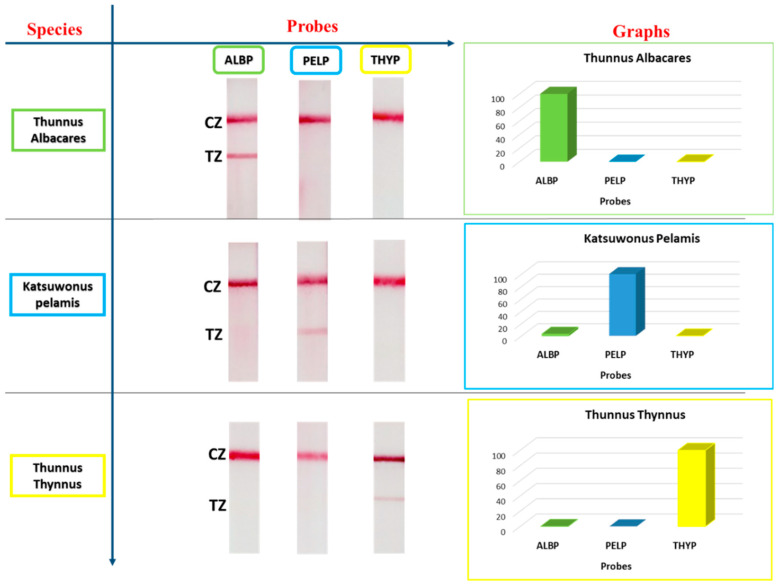
Specificity studies. Amounts of 100 fmol amplification product of each examined species were hybridized, separately, with all three probes and detected by the proposed sensing device. The left panel presents the images of the sensing membranes. The right panel presents the diagrams of the test zone color intensity obtained from the sensor for each hybridization product. TZ: test zone; CZ: control zone.

To evaluate the probe specificity, 100 fmol of each PCR product was hybridized separately with all three species-specific probes and applied to the sensor. The strips were then scanned by a regular scanner, and the color intensity of the test zones of the strips was measured using the online image processing software, Image J. (1.54g) The results are presented in Figure 4. We observed that each PCR product gave a positive result only with its complementary probe. Thus, the proposed method has high specificity for the three tuna species studied. The method can efficiently detect the examined sample without cross-reactivity from the other tuna species.

### 3.4. Precision

**Figure 5:**
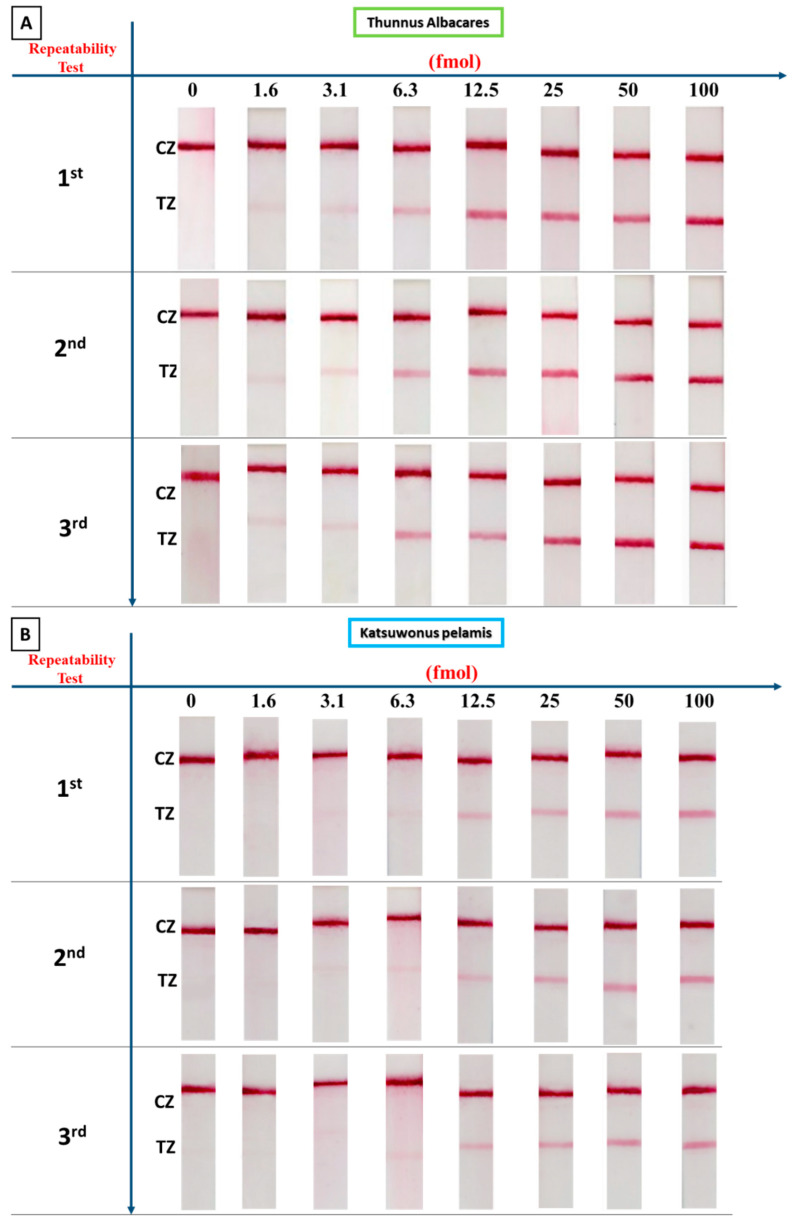

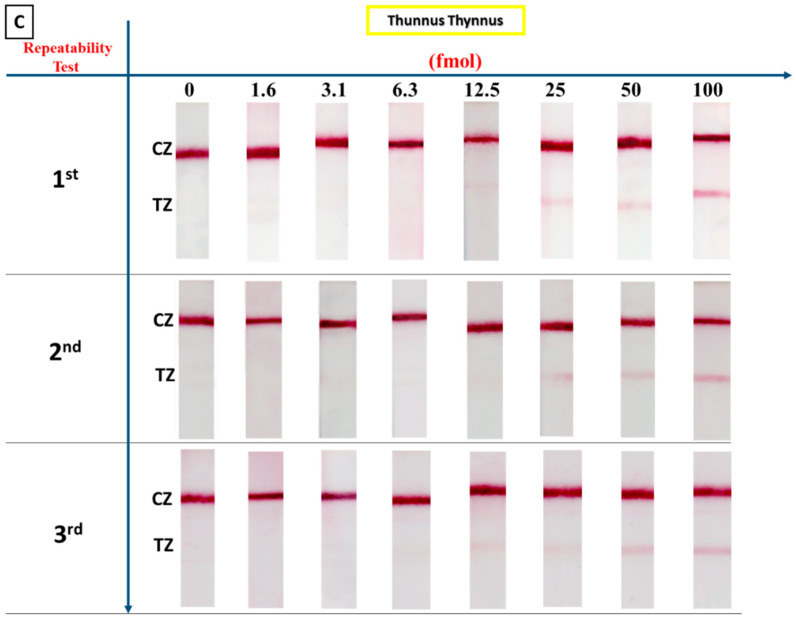
Precision of the sensing device for (**A**) *T. albacares*, (**B**) *K. pelamis*, and (**C**) *T. thynnus*. TZ: test zone; CZ: control zone.

Triplicate analysis with the sensing device of PCR products (from fresh tissue) from each tuna species was performed to evaluate the repeatability of the assay. DNA was isolated from each fish tissue from each species and subjected to PCR. Each PCR product was analyzed in triplicate with the sensor. Figure 5 shows the results of the repeatability for each type of tuna fish. As observed, the proposed sensing device provides high repeatability for all tuna species.

Figure 5 shows the results of three experiments carried out for each tuna species. The results were processed with Image J software (1.54g) to calculate the coefficients of variation (CVs) of the signal at the test zones of the strips for each tuna species. For the lowest amount detected with the naked eye, the %CVs were equal to 1.2% for 1.6 fmol of *T. albacares*, 1.2% for 3.1 fmol of *K. pelamis*, and 0.8% for 12.5 fmol of *T. thynnus*.

### 3.5. Application of the Method to the Detection of Fish Adulteration in Heat-Processed Fish Mixtures

**Figure 6:**
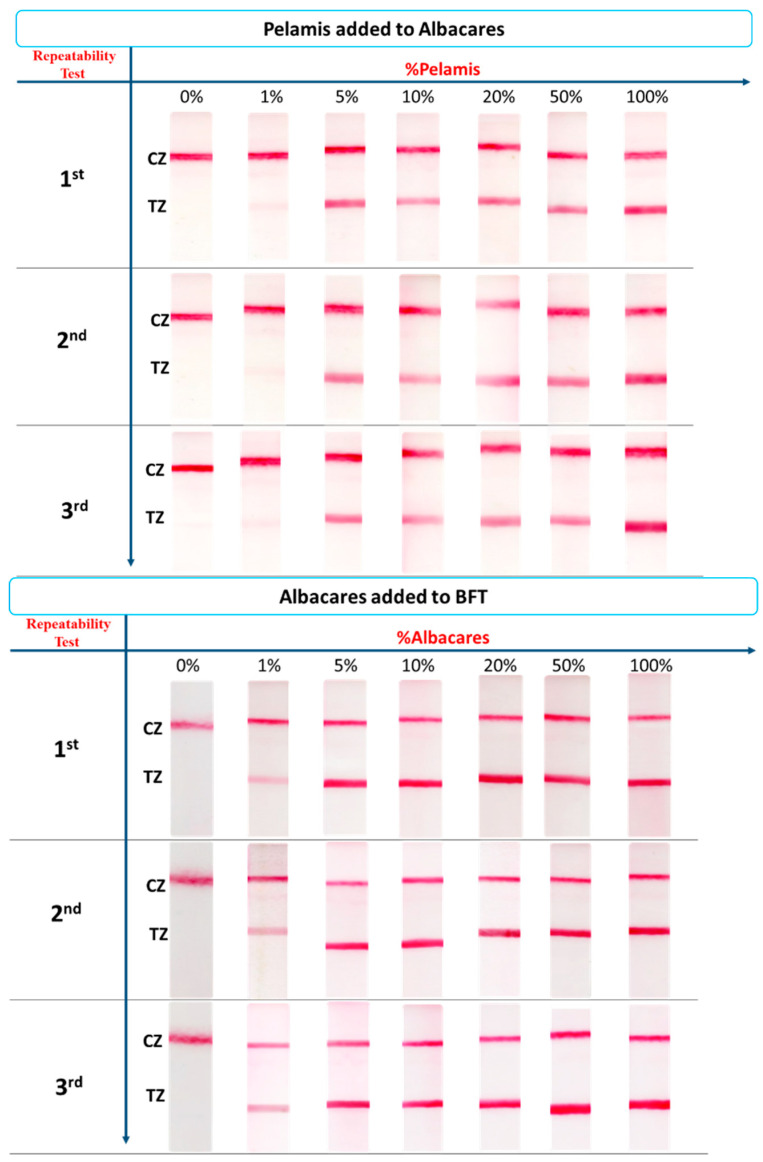

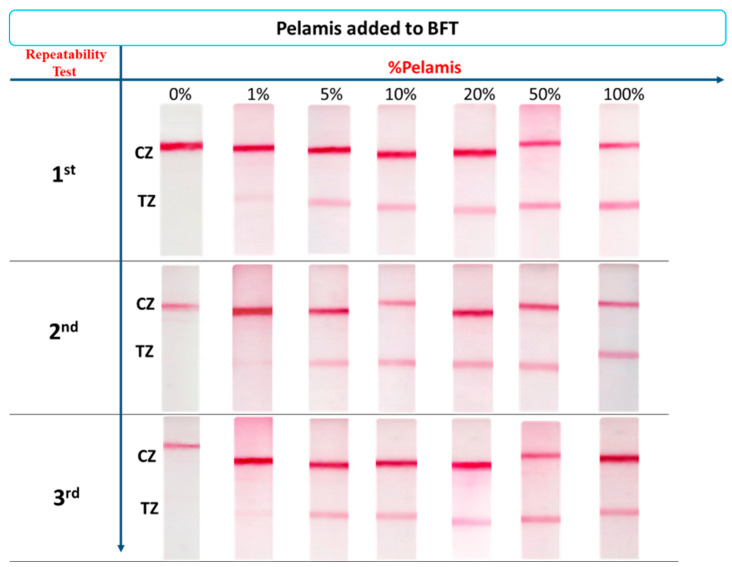
Tuna fish adulteration detection in binary heat-processed mixtures (simulated canned products) using the proposed sensing device. The adulterant is present at a range of 1 to 100% in the binary mixtures. TZ: test zone; CZ: control zone. BFT: Bluefin tuna or *Thunnus thynnus*.

The final evaluation of the developed method involved the application to processed (heat-treated) fish mixtures for tuna adulteration detection. In the market, tuna products of high value (e.g., *T. thynnus*) are usually adulterated with lower-priced tuna species, such as *T. albacares* and *K. pelamis*. Thus, *T. albacares* adulteration with *K. pelamis* and *T. thynnus* adulteration with *K. pelamis* or with *T. albacares* were selected as the three model cases for experimentation. Home-made canned products were prepared by mixing each fish tissue of the three tuna species with salt, pepper, paprika, onion, oil, vinegar, and tomato followed by frying and boiling, thereby simulating canned conditions. DNA was then isolated from the home-made canned products. Binary mixtures were prepared using the isolated DNA in various percentages of adulteration that ranged from 1 to 100%. A total mass of 25 ng of DNA of each mixture was subjected to PCR amplification. The PCR products were detected with the sensing device. For *T. thynnus*–*T. albacares* and *T. thynnus*–*K. pelamis* mixtures, a 5-fold dilution of the PCR product was carried out prior to the application to the sensing device. The PCR products from *T. albacares*–*K. pelamis* mixtures were used undiluted. Figure 6 presents the results from tuna fish adulteration detection in various binary mixtures at an adulteration range of 1–100%. Each test was repeated three times.

It is observed that as low as 1% of *K. pelamis* in *T. albacares* or in *T. thynnus* and 1% of *T. albacares* in *T. thynnus* were detectable with the naked eye using the proposed molecular rapid test in binary mixtures of isolated DNA from home-made cans of the three tuna species. The coefficient of variations %CVs (*n* = 3) was ranged between 1.5 and 3.0%.

## 4. Optimized Protocols and Methods

### 4.1. DNA Isolation from Fish Tissue

Prior to DNA isolation, the tissue was rinsed with water and ethanol and dried using absorbent paper. The NucleoSpin Tissue kit was used according to the manufacturer’s instructions. The tissue was homogenized well with proteinase K and lysis buffer before starting the isolation procedure. The concentration of the isolated DNA was determined by obtaining the absorbance values at 260/280 nm using the DeNovix spectrophotometer.

### 4.2. Polymerase Chain Reaction (PCR)

The PCR was performed in a final volume of 25 μL. The PCR master mix contained the following: 1×KAPA2G Buffer A, 2.5 mM MgCl_2_, 0.2 mM of a dNTP mixture containing dATP, dGTP, dCTP, and dUTP, 0.5 μM of Forward and Reverse primer of each tuna species, respectively, 0.5 U KAPA 2G Fast DNA Polymerase, and 10–100 ng of isolated DNA. The PCR steps included an initial denaturation step at 98 °C for 3 min and 30 cycles of denaturation at 98 °C for 10 s, annealing at 62 °C for 30 s, and extension at 72 °C for 30 s. The final extension step was performed at 72 °C for 10 min.

### 4.3. Preparation of Conjugated Gold Nanoparticles with Anti-Biotin Antibody

A 1-mL volume of the AuNP solution (0.15 nmoL/L) was added to a silanized microcentrifuge tube, and the pH was adjusted to 9.0 by adding 200 mM borax solution. The anti-biotin antibody solution was prepared by adding 4 μL of 1 g/L anti-biotin antibody to 400 μL of 2 mM borax. The anti-biotin antibody solution was added to the previous gold solution gradually by stirring. The mixture was incubated at an ambient temperature for 45 min in the dark. Then, 100 μL of 100 g/L BSA in 20 mM borax was added, and the solution was incubated for 10 min at an ambient temperature in the dark. Afterwards, centrifugation at 4500× *g* for 15 min was performed, and the supernatant was discarded. The pellet was redispersed in 500 μL of 10 g/L BSA in 2 mM borax. A second centrifugation at 4500× *g* for 7 min was also conducted, and the isolated red pellet was redispersed in 100 μL of 1 g/L BSA and 1 g/L NaN_3_ in 2 mM borax. The final conjugated gold nanoparticles were stored at 4 °C for further use. A 5-μL volume of conjugated nanoparticles was deposited onto the conjugation pad of the sensing device.

### 4.4. Construction of the Sensing Device

Reagents were dispensed onto the nitrocellulose membrane of the sensor using a proper syringe and the Linomat 5 TLC applicator (Camag). The control zone consisted of 1.5 ng of biotinylated Bovine Serum Albumin (b-BSA), and the test zone consisted of 10 pmol of polydT-dT_30_. The diluent of b-BSA was 5% (*v*/*v*) MeOH, 2% (*w*/*v*) sucrose, and 1×PBS pH 7.4. The polydT-dT_30_ was diluted properly with 5% MeOH (*v*/*v*), 2% sucrose (*w*/*v*), and 6×SSC pH 7.0. The volumes of the above solutions were adjusted according to the number of strips prepared. After the spraying of the solutions, the membrane was placed into the UV-Crosslinker for 15 min at 125 mJ/cm^2^ for reagent immobilization. The strips (4 mm × 70 mm) consisted of a plastic support, on which the individual parts, the membrane, the conjugation pad, and the blotting pads were assembled.

### 4.5. Molecular Rapid Test for Visual Detection of Tuna Species

Firstly, a hybridization reaction was performed between the PCR products and their complementary polydA-probes. A 1.5-μL volume of each PCR product was mixed with 1.5 μL of 0.5 μM of the polydA-probe. Then, denaturation of the PCR product was performed by adding 1.5 μL of 0.4 M NaOH in the above solution, followed by incubation for 5 min at an ambient temperature. Neutralization of the solution was followed by adding 1.5 μL of a solution which contained 0.4 M Tris-base pH 7.6 and 0.4 M HCl. Incubation for 10 min at 37 °C was followed for the hybridization reaction to be completed. For the detection of tuna species with the molecular rapid test, 5 μL of the above hybridization solution was added onto the conjugation pad, and the strip was immersed in a tube that contained 300 μL of 1×PBS, 1% (*v*/*v*) Glycerol, 1% (*v*/*v*) Tween, 1% (*w*/*v*) BSA, and 0.75% (*w*/*v*) SDS. The signal was observed after 10–15 min.

## 5. Conclusions

In this work, we have developed a new molecular rapid test for the DNA-based detection of tuna fish adulteration with the naked eye. This is the first time that a DNA sensor has been developed for tuna adulteration detection. The three most common tuna species, namely *Katsuwonus pelamis*, *Thunnus albacares*, and *Thunnus thynnus*, were visually identified using a rapid test at low levels and gold nanoparticles as reporters. The method included DNA isolation from fish tissue, amplification of species-specific DNA sequences by PCR, and visual detection with the naked eye using the sensor. The method was successfully applied to fresh fish tissue samples, as well as heat-treated and cooked fish samples, in the presence of other food ingredients simulating canned conditions. The method was finally applied for the detection of tuna adulteration in binary home-made canned mixtures with adulteration percentages ranging from 1 to 100%. As low as 1% of adulteration was detected with the naked eye using the proposed sensor with high repeatability and specificity. The developed molecular rapid test is easy to construct, simple to use, and provides excellent analytical performance. A repeatability study provided %CVs in the range of 0.8–3.0%, proving the excellent repeatability of the proposed rapid test. The sensing device can be provided in the future as a ready-to-use device in the form of a dry-reagent device.

## Figures and Tables

**Table 1:** Sequences of primers and probes used in the present work.

Species	Targeted Gene	Name of Primer and Probes	Sequences (5′→3′)	Size of Amplicon (bp)	Reference
Yellowfin tuna— *Thunnus albacares*	D-loop region	Forward primer (ALBF)	CGAGATTTAAGACCTACCATAACAAC	85	[1]
		Reverse primer (BALBR)	Biotin-TGCGCTTAAATTTACCTGACTT		
		Probe (ALBP)	TCGTCTAAGCCATACCAAGTATCCC		
Skipjack tuna— *Katsuwonus pelamis*	Cytb	Forward primer (PELF)	GGTCCTAGCTCTTCTTGCA	238	[2]
		Reverse primer (BPELR)	Biotin-TGCAAGTGGGAAGAAGATG		
		Probe (PELP)	CCCTTCATTATCATCGGCCA		This study
Atlantic Bluefin— *Thunnus thynnus*	NADH5	Forward primer (THYF)	AACTCTTTATCGGGTGGGAG	200	[2]
		Reverse primer (BTHYR)	Biotin-AGCGGTTACGAACATTTGCTTC		
		Probe (THYP)	CTGCTCTACAAGCGGTAGTA		This study

## Data Availability

Data are available upon requirement.

## Supplementary Materials

The following supporting information can be downloaded at: https://www.mdpi.com/article/10.3390/bios14020082/s1. Figure S1: Electropherogram of PCR products of the three different tuna species. The DNA Marker ΦΧ174 HaeIII digest was used for qualitative analysis. (P: *Katsuwonus pelamis*, A: *Thunnus albacares* and T: *Thunnus thynnus*); Figure S2: Electrophoresis in 2% agarose gel of the three PCR reactions performed in three different annealing temperatures. The DNA Marker ΦΧ174 HaeIII digest was used for qualitative and quantitative analysis. P: positive, N: Negative and (xx) the annealing temperature; Figure S3: Electrophoresis in 2% agarose gel of the three PCR reactions performed in three different cycles: 35, 30 & 25 cycles. The DNA Marker ΦΧ174 HaeIII digest was used for qualitative and quantitative analysis, Figure S4: Optimization of the hybridization temperature of the PCR product to species-specific probe. An amount of 100 fmol of the PCR product for *K. pelamis* was hybridized to its specific probe at A. 37 °C and B. 42 °C. TZ: Test Zone and CZ: Control Zone; Figure S5: Optimization of the concentration of species-specific probes in the hybridization reaction prior to the application to the sensing device. An amount of 6.3 fmol of the PCR product of K. pelamis was hybridized to its specific probe in three different concentrations: A. 0.5 pmol/μL, B. 1 pmol/μL, C. 2 pmol/μL. (TZ: Test Zone and CZ: Control Zone); Table S1: DNA concentration after DNA isolation from fish samples using various kits.
