# Supplementary material for: Molecular Rapid Test for Identification of Tuna Species
Source: Biosensors (Basel). 2024 Feb 2;14(2):82. doi: 10.3390/bios14020082 (PMC10887179; doi:10.3390/bios14020082)
Supplement: Supplementary file 1 [file biosensors-14-00082-s001.zip › biosensors-2820544-supplementary.pdf]

Supporting Information

Molecular Rapid Test for Identification of Tuna Species

Isidora P. Gkini[1], Panagiotis Christopoulos[1], Alexis Conides[3], Despina P. Kalogianni[1,]* and Theodore K. Christopoulos[1,2,]*

[1] Analytical/Bioanalytical Chemistry & Nanotechnology Group, Department of Chemistry, University of Patras, Patras, Greece 26504

[2] Institute of Chemical Engineering Sciences / Foundation for Research and Technology Hellas (FORTH/ICE-HT), Patras, Greece 26504

[3] Hellenic Centre for Marine Research, Institute for Marine Biological Resources, 46.7 km Athens-Sounion, Mavro Lithari, Anavyssos, Attika, Greece 19013

Corresponding authors

Theodore K. Christopoulos, E-mail: tchrist@upatras.gr

Despina P. Kalogianni, E-mail: kalogian@upatras.gr

DNA isolation

DNA was isolated from fish samples using three different DNA isolation kits. After isolation, the absorbance of the samples was measured at 260 nm and the results are presented in the following Table.

Table S1: DNA concentration after DNA isolation from fish samples using various kits.

**Tuna species**	NucleoSpin Tissue (Macherey-Nagel)	Genomic DNA Isolation Kit (Norgen Biotek)	NucleoSpin Plant II (Macherey-Nagel)
	Concentration (ng/uL)	Concentration (ng/uL)	Concentration (ng/uL)
T. albacares	97.0	n.d. (Too Low)	-
K. pelamis	80.5	n.d. (Too Low)	15.0
T. thynnus	238	24.8	-

*n.d.: not detected

Electrophoresis of PCR products

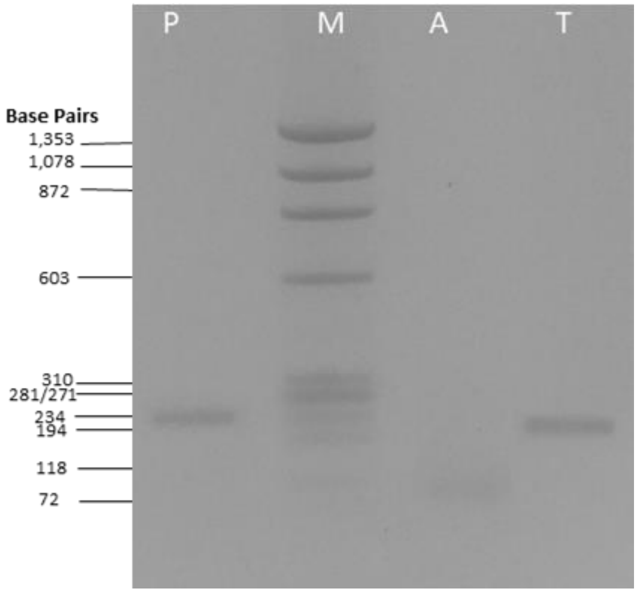

Figure S1. Electropherogram of PCR products of the three different tuna species. The DNA Marker ΦX174 HaeIII digest was used for qualitative analysis. (P: *Katsuwonus pelamis*, A: *Thunnus albacares* and T: *Thunnus thynnus*).

The three PCR products had a size of 85 bp for *Thunnus albacares*, 238 bp for *Katsuwonus pelamis* and 200 bp for *Thunnus thynnus.*

Optimization of primers annealing temperature in PCR

Three different primers annealing temperatures (50, 55, and 62 ^oC) were tested in the PCR using DNA isolated from *K. pelamis* tissue. The PCR products were then electrophorized in 2% agarose gel using ethidium bromide for staining. The electropherogram is presented in the following Figure.

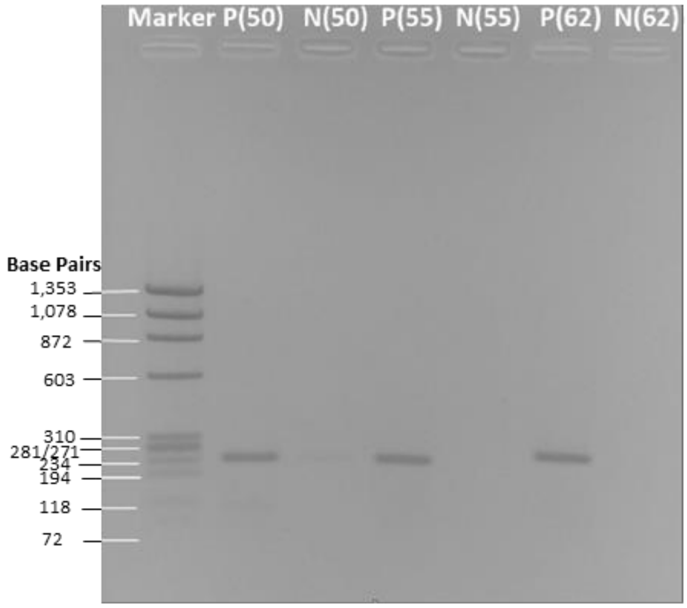

Figure S2. Electrophoresis in 2% agarose gel of the three PCR reactions performed in three different annealing temperatures. The DNA Marker ΦX174 HaeIII digest was used for qualitative and quantitative analysis. P: positive, N: Negative and (xx) the annealing temperature.

The quantification of the PCR products for *K. pelamis* DNA sequence (238 bp) was performed using the software Image J and the Gel Analyzer tool. The results showed that the yield of the PCR reaction was 269.90 fmol/uL when the annealing temperature was 50 ^oC, 269.63 fmol/uL when the temperature was set at 55 ^oC and the highest yield of 299.86 fmol/uL was obtained at 62 ^oC.

Optimization of PCR cycles

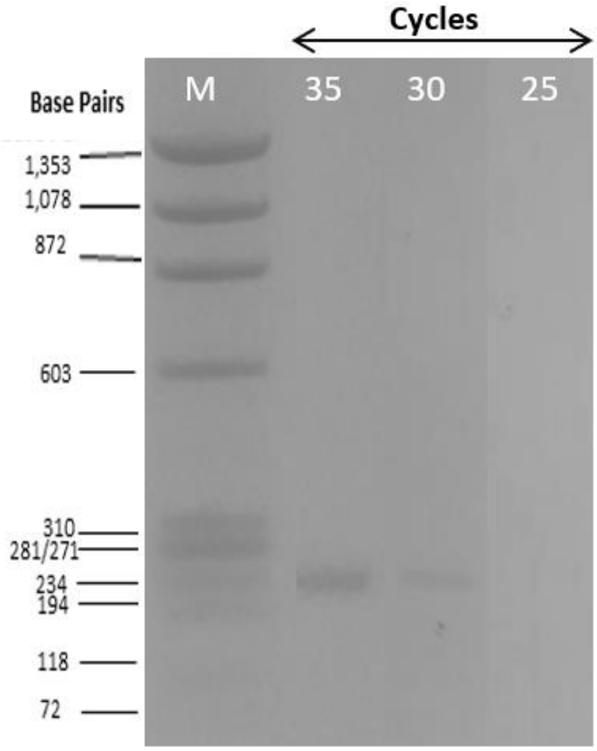

Figure S3. Electrophoresis in 2% agarose gel of the three PCR reactions performed in three different cycles: 35, 30 & 25 cycles. The DNA Marker ΦX174 HaeIII digest was used for qualitative and quantitative analysis.

An amount of 100 ng of isolated DNA was used in each reaction. The quantification of the PCR products for *K. pelamis* DNA sequence (238 bp) was performed using the software Image J and the Gel Analyzer tool. As observed in Figure S3, no PCR product was produced in 25 cycles.

Optimization of the hybridization temperature to the species-specific probes

Two hybridization temperatures, 37 °C and 42 °C, were tested for the hybridization of 100 fmol of PCR product for *K. pelamis* to its specific probe prior to the application to the sensing device. The results are shown in Figure S3.

According to Figure S4, there is no significant difference in the signal between the two hybridization temperatures. However, the temperature of 37 °C was chosen, as the signal at the test zone of the strip was slightly stronger.

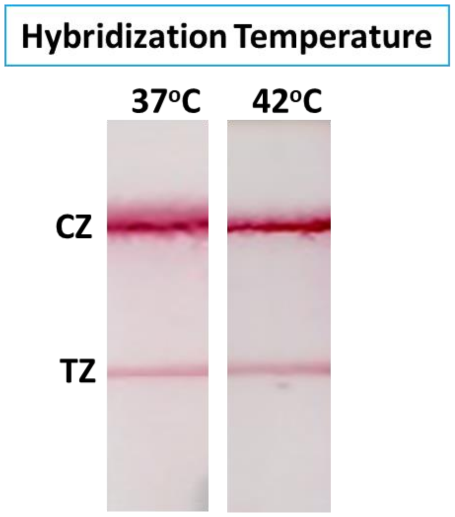

Figure S4. Optimization of the hybridization temperature of the PCR product to species-specific probe. An amount of 100 fmol of the PCR product for *K. pelamis* was hybridized to its specific probe at **A**. 37 °C and **B**. 42 °C. TZ: Test Zone and CZ: Control Zone.

Optimization of the concentration of species-specific probe

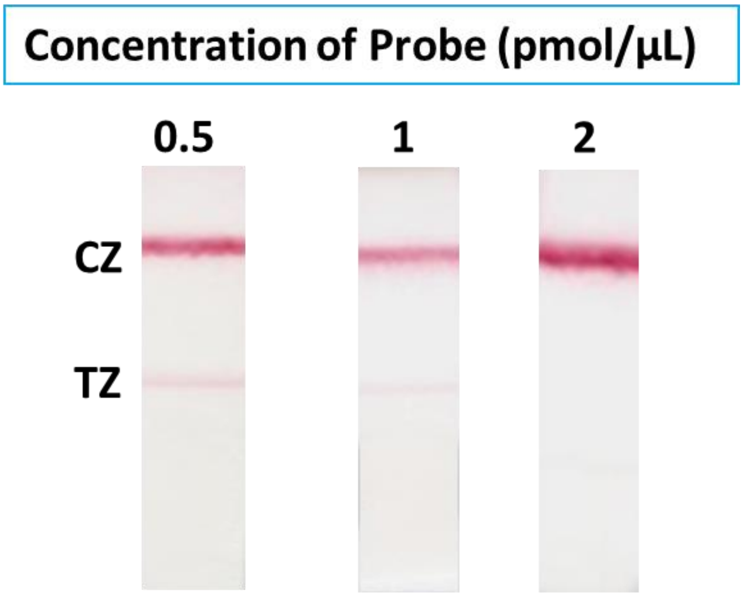

Figure S5. Optimization of the concentration of species-specific probes in the hybridization reaction prior to the application to the sensing device. An amount of 6.3 fmol of the PCR product of *K. pelamis* was hybridized to its specific probe in three different concentrations: **A**. 0.5 pmol/μL, **B**. 1 pmol/μL, **C**. 2 pmol/μL. (TZ: Test Zone and CZ: Control Zone).

As observed in Figure S5, the optimum concentration of the specific probe is 0.5 pmol/μL, because it provided the most intensive signal at the test zone of the strip.
